# Monosodium L-glutamate and fats change free fatty acid concentrations in intestinal contents and affect free fatty acid receptors express profile in growing pigs

**DOI:** 10.29219/fnr.v63.1444

**Published:** 2019-07-17

**Authors:** Yun Su, Zemeng Feng, Yumin He, Lingling Hong, Gang Liu, Tiejun Li, Yulong Yin

**Affiliations:** 1 Hunan international joint laboratory of Animal Intestinal Ecology and Health, Laboratory of Animal Nutrition and Human Health, College of Life Sciences, Hunan Normal University, Changsha, China; 2 Key Laboratory of Agro-ecological Processes in Subtropical Region, Institute of Subtropical Agriculture, Chinese Academy of Sciences; National Engineering Laboratory for Pollution Control and Waste Utilization in Livestock and Poultry Production; Hunan Provincial Engineering Research Center for Healthy Livestock and Poultry Production; Scientific Observing and Experimental Station of Animal Nutrition and Feed Science in South-Central, Ministry of Agriculture, Changsha, China; 3 Hunan Co-Innovation Center of Animal Production Safety, CICAPS, Changsha, China; 4 Guangdong Wangda Group Academician Workstation for Clean Feed Technology Research and Development in Swine, Guangdong Wangda Group Co., Ltd, Guangdong, China; 5 Hunan Co-Innovation Center of Safety Animal Production, College of Animal Science and Technology, Hunan Agricultural University, Changsha, China

**Keywords:** monosodium L-glutamate, fat, free fatty acid, intestinal luminal, free fatty acids receptors, lipid metabolism

## Abstract

**Background:**

Obesity and its related metabolic syndrome continue to be major public health problems. Monosodium L-glutamate (MSG) may cause metabolic diseases such as obesity. Meanwhile, the Chinese population has undergone rapid transition to a high-fat diet. There is little information available on the effect of MSG and fat alone, or in combination, on free fatty acids (FFAs), lipid metabolism and FFA receptors.

**Objective:**

The aim of this study was to evaluate the effects of MSG and fat alone, or in combination, on intestinal luminal FFAs and expression of gastrointestinal FFA receptors. The aim was also to test whether dietary fat and/or MSG could affect expression of genes related to fatty acid metabolism.

**Design:**

A total of 32 growing pigs were used and fed with four iso-nitrogenous and iso-caloric diets. Pigs in the four treatments received diets with one of two fat concentrations levels (4.4 and 9.4%) and one of two MSG dose levels (0 and 3%), in which most of the fat were brought by soybean oil. The concentration of short chain fatty acids (SCFAs) in cecum and colon, long chain fatty acids (LCFAs) in ileum, cecum and colon, and FFAs receptors expression in hypothalamus and gastrointestinal tract were determined.

**Results:**

MSG and/or fat changed intestinal luminal SCFAs, levels of LCFAs, and showed an antagonistic effect on most of LCFAs. Simultaneously, MSG and/or fat decreased the expression of FFA receptors in hypothalamus and gastrointestinal tract. MSG and/or fat promoted fat deposition through different ways in back fat.

**Conclusion:**

Our results support that MSG and/or fat can alter intestinal luminal FFAs composition and concentration, especially LCFAs, in addition, the expression of FFA receptors in ileum and hypothalamus could be decreased. Moreover, MSG and/or fat can promote protein deposition in back fat, and affect the distribution and metabolism of fatty acids in the body tissues and the body’s ability to perceive fatty acids; these results provide a reference for the occurrence of fat deposition and obesity caused by high-fat and monosodium glutamate diet.

## Popular scientific summary

- MSG and/or fat changed intestinal luminal SCFAs and LCFAs concentration, with generally antagonistic effects on most of LCFAs.
- MSG and/or fat decreased the expression of FFAs receptors in hypothalamus and gastrointestinal tract.
- MSG and/or fat affected the distribution and metabolism of fatty acids in the body tissues and the body’s ability to perceive fatty acids.

Obesity and its related metabolic syndrome including type II diabetes mellitus (1) and cardiovascular diseases (2) are major public health problems in the developed countries (3–5). The primary reason for obesity is the excess intake of energy, which is stored in the form of triglycerides (6, 7). As energy supplier and important building blocks of adipogenesis, free fatty acids (FFAs) play a critical role in energy homeostasis and pathogenesis of obesity and related syndrome (8–10). FFAs can be divided into short chain fatty acids (carbon chain <6, SCFAs) and long chain fatty acids (LCFAs). SCFAs are mainly produced by anaerobic bacterial fermentation which degrades complex carbohydrates in distal intestine (11), while LCFAs are mainly produced by transformation from neutral fat, cholesterol ester and phospholipids in liver. These intestinal luminal FFAs are absorbed into blood circulation via passive diffusion and/or fatty acid transporters by flip-flop mechanism (12, 13). Evidences have demonstrated that, except for primarily used as energy source for enterocytes (14), FFAs such as SCFAs could enhance intestinal squirm and ion transportation via releasing of 5-hydroxytryptamine (15–17), increase the intestinal microbiota diversity, decrease the colonisation of hazardous bacteria (18–20) and regulate intestinal immunity (21). The effect of FFAs is finely regulated by fatty acid receptors, which subsequently activate downstream signalling cascade, and finally affecting host physiological as well as immune function (22). The most extensively identified FFAs receptors belong to G protein-coupled receptor (GRP) and mainly include GPR40, GPR41, GPR43, GPR84, GPR119 and GPR120 (23–26). Many compelling investigations had shown that FFAs receptors were related with lipid metabolism, immunity and pathogenesis of obesity, chronic inflammation and diabetes (27, 28). In fact, FFA receptors are regarded as therapeutic targets for metabolic disorders (29). Considering the importance of FFAs and FFA receptors, it is important to investigate luminal FFAs concentration and gastrointestinal FFA receptors.

Monosodium L-glutamate (MSG) is widely used as a flavour enhancer with an umami taste, especially in Asian countries including China (30, 31). MSG demand is still increasing in the world for the pleasant taste it brings; however, the controversy regarding MSG safety continuously exists since a report that described the so-called Chinese restaurant syndrome in 1968 (32, 33). Furthermore, many studies have demonstrated that MSG leads to metabolic diseases such as obesity and diabetes through insulin resistance (34, 35), hypothalamic lesions and leptin resistance (36), or alters hepatic gene expression of nitrogen and lipid metabolism (37). Notably, with the industrial and social development, the Chinese population, including children and adolescents, has undergone a rapid transition to a high-fat diet. As we know, the main reason of obesity is the excessive deposition of fat, and the amount of body fat content depends on the processes of fat synthesis and decomposition. Excess fat consumption makes it easy to induce obesity (38). Most of the fatty acids required for body fat deposition in animals come from the whole process synthesis of fatty acids, during which the synthesis of triglycerides (TAG) catalyses by fatty acid synthase (FAS) with acetyl-coa and malonyl-coa. Excessive expression level of FAS can significantly increase the deposition of triglycerides in the body, thus leading to obesity (39–41). Besides, hydrolysis of animal fat is mainly catalysed by hormone-sensitive lipase, which is a rate-limiting enzyme for triglyceride degradation in fat cells and plays an important role in regulating energy balance (42). However, the impacts of MSG and fat alone, or in combination, on fat metabolism in adipose tissue, intestinal luminal FFAs metabolism and gastrointestinal FFA receptors are rarely reported. Recently, researches aimed to reveal the association between flavour enhancer and metabolism have shown that flavour enhancer affects body metabolism by altering intestinal microbiota (43). Dietary MSG and/or fat alter intestinal microbiota by enhancing the colonisation of energy-harvesting microbes (44). As the majority of luminal FFAs are produced by bacterial fermentation, we hypothesise that MSG and/or fat could change the intestinal luminal FFAs metabolism and gastrointestinal FFA receptors expression. Pig is a suitable model for studying human nutrition because its nutritional and digestive characteristics are similar to those of humans (45). Thus, the present study was conducted to investigate the effect of MSG and fat alone, or in combination, on fat metabolism in adipose tissue, FFA concentrations in intestinal luminal contents and the expression profile of FFA receptors in hypothalamus and gastrointestinal tract of growing pigs.

## Materials and methods

All experimental procedures used in this study were approved by the Animal Care and Use Committee of the Chinese Academy of Sciences.

### Experiment design

A total of 32 growing pigs (York × Landrace × Duroc, average body weight 25.0 ± 1.3 kg) from four litters were randomly divided into four groups (eight repeats); the percentage of males and females was fifty-fifty. In the present study, 2 × 2 factorial design was used. Four iso-nitrogenous and iso-caloric diets (basal diet [BD]; high fat diet [HF]; basal diet with 3% MSG [BDM]; and high fat diet with 3% MSG [HFM]) were provided to growing pigs; most of the fat were brought by soybean oil. BD group was used as control. The detailed fatty acid compositions and nutrient level of the four diets are shown in Table 1. The diets and water were provided to the pigs freely. The feeding lasted for 30 days; blood samples from jugular vein were collected into heparin-coated tubes and then centrifuged (3,000 × g for 10 min at 4°C). The supernatants (plasma) were immediately stored at −80°C until analysis. All pigs were sacrificed by jugular puncture under general anaesthesia, via intravenous injection of 4% sodium pentobarbital solution (40 mg/kg body weight [BW]). Samples from the hypothalamus, back fat and different segments of gastrointestinal tract including stomach, duodenum, jejunum, ileum and colon (cleaned by ice-cold saline before sampling) were collected immediately and then frozen in liquid nitrogen and stored at −80°C until analysis. The contents of ileum, cecum and colon were collected and then immediately stored at −20°C until analysis. The back fat sample was preserved in neutral formalin.

**Table 1:** Composition of experimental diets

Item	BD	HF	BDM	HFM
Ingredient composition (%)				
Corn	71.37	59.80	70.30	59.58
Soybean meal	19.20	21.27	16.80	21.50
Corn starch	0.00	7.00	0.00	5.00
Corn Gluten Meal	5.00	2.50	7.00	3.10
MSG	0.00	0.00	3.00	3.00
Alanine	1.58	1.58	0.00	0.00
L-Lysine monohydrochloride	0.15	0.15	0.20	0.12
Soybean oil	0.00	5.00	0.00	5.00
Premix^a^	2.70	2.70	2.70	2.70
Calculated nutrient level				
Digestible energy (MJ/kg)	13.98	13.92	13.87	13.98
Crude protein (%)	17.93	17.88	17.95	17. 91
Ether extract (%)	4.35	9.39	4.51	9.45
Ca (%)	0.60	0.59	0.58	0.59
*P* (%)	0.45	0.48	0.44	0.46
Lys (%)	0.83	0.85	0.83	0.83
Met (%)	0.26	0.25	0.28	0.25
Thr (%)	0.56	0.55	0.56	0.55
Fatty acid composition (%)^b^				
Myristic acid	0.13	0.10	0.17	0.11
Palmitic acid	15.18	12.26	15.39	12.14
Palmitoleic acid	0.19	0.14	0.22	0.13
Heptadecanoic acid	0.22	0.21	0.25	0.21
Stearic acid	3.19	3.98	3.45	4.01
Oleic acid	21.47	23.47	21.10	23.31
Linoleic acid	54.22	52.81	54.00	52.53
Arachidic acid	0.42	0.43	0.42	0.42
Eicosenoic acid	0.27	0.72	0.27	0.74
α-Methyl linolenate	3.30	4.90	3.43	4.99
Behenic acid	0.24	0.43	0.25	0.43
Tetracosanoic acid	0.78	0.52	0.77	0.54

a Composition (%): CaHPO4, 27.78; Mountain Flour, 24.07; NaCl, 11.11; medicalstone, 12.33; powdered rice hulls, 18.81; FeSO_4_, 0.74; ZnSO_4_, 0.74; selenium powder (1%), 0.15; iodine powder (1%), 0.15; CuSO_4_, 0.37; MnSO_4_, 0.30; choline chloride, 2.22; growth pig multidimensional, 1.11; antioxidants (ethoxyquin 66%), 0.11.

b The contents of fatty acid were all measured values.

### Morphology of back adipose tissue

The solution on the fat tissue was dried with filter paper, frozen and fixed with optimal cutting temperature (OCT) embedding agent and then frozen and sliced in Leica CM1950 (Leica Microsystems Nussloch GmbH, Heidelberger, Germany). Adipocytes of back adipose tissue were observed under ordinary light microscope after stained with haematoxylin-eosin and sealed with gelatin (Micrometrics TM; Nikon Eclipse E200, Tokyo, Japan).

### Measurement of total FFA concentrations in plasma and hypothalamus

Total FFA concentrations in plasma and hypothalamic homogenate were measured using total FFAs enzyme-linked immune sorbent assay (Elisa) kit following the manufacturer’s instructions (CUSBIO, Wuhan, China).

### Measurements of concentration of SCFAs in intestinal luminal contents

All the contents of cecum and colon were lyophilised and then weighted. About 0.5g of lyophilised samples was dissolved with 2 mL sulfuric acid (2%, v/v), vortex mixed and then centrifuged at 10,000 g for 10 min at 4 ºC; 1 mL supernatant and 0.25 mL metaphosphoric acid (25%, v:v = 3:1) were fully mixed. Then the mixture was added into 10 mL centrifuge tube with 2 g dried acid adsorbent (anhydrous sodium sulphate: 50% sulfuric acid: diatomite = 35:1:15), and added 3 mL chloroform subsequently, vortex fully. After clarified transparently, centrifuged at 10,000 g for 15 min at 4 ºC, finally at least 600 μl supernatant was used for analysis. The standard solution of SCFAs (acetate, propionate, butyrate, isobutyrate, valerate and isovalerate) was prepared by mixing 1 mL standard stock solution (100 μl corresponding FFAs solution was diluted with chloroform to 100 mL, respectively) with 9 mL chloroform. The concentration of FFAs was determined by gas chromatography (GC)-electrospray ionisation (Agilent, 6890). The capillary columns (1.82 m × 0.2 mm) were filled with 80/100 red diatomite (HP Inc, USA). The results were presented with mg SCFA/g lyophilized sample.

### Measurement of LCFAs concentrations in diets and intestinal contents

The LCFAs of each content in diets, ileum, cecum and colon were extracted with mixture solution (petroleum ether: benzene = 1:1) and then were methyl esterified with methanol solution (4 mol/L). The concentrations of LCFAs were determined by LC/mass spectrometer (HPLC Ultimate 3000, Dionex; 3200 Q TRAP LC-MS/MS, AB) with GC using a capillary column (HP-INOWAX, 30 m × 2.5 mm × 2.5 μm) and F.A.M.E. Mix, C4-C24 analytical standard, wt. % (varied) (Sigma-Aldrich). The program of LC/mass spectrometer was as follows: initial temperature: 150°C, 3 min; then raised to 200°C in a 8 °C/min speed, 1 min; finally raised to 250°C in a 15°C/min speed, 4 min. The temperature of sample injector and detector was at 240°C and 260°C respectively, with hydrogen as gas carrier in a 40 mL/min flow rate. Identification of individual LCFAs was performed by comparisons with authentic standard mixtures. The results were presented with the percentage of individual LCFAs to total FFAs.

### Real-time transcription polymerase chain reaction (RT-PCR)

Total RNA was isolated from stored samples with TRIZOL regent (Invitrogen, USA) and treated with DNase I (Invitrogen, USA) according to the manufacturer’s instructions. For each sample, the RNA quality was checked by 1% agarose gel electrophoresis, stained with 10 µg/mL ethidium bromide. Synthesis of the first strand cDNA was performed with primers (mix of random primer and oligo dT primer, 1:1) and PrimeScript^®^ 1st Strand cDNA Synthesis Kit (TaKaRa, Dalian, China) according to the manufacturer’s instructions.

The primers used in the present study were designed using Primer 5.0 software, and the detail information was listed in Table 2. β-actin was used as the reference gene to normalise target gene transcript levels. The method for real-time PCR performing and data analysis was referred to previous study (46).

**Table 2:** Primers used in this study

Gene	Provenance	Sequence	Length
*β-actin*	XM_003357928.1	F: GGACTTCGAGCAGGAGATGG	233 bp
		R: GCACCGTGTTGGCGTAGAGG	
*GPR40*	XM_003127043	F: TGCTCTGACCTCCTGCTGG	89 bp
		R: CACACACCCCCCAGGAATAG	
*GPR41*	NM_005304.3	F: GCTGCTGTTCCTGCCTTTC	98 bp
		R: TGAAGAAGATGAATCCAGAGAGTG	
*GPR43*	XM_003127046.1	F: CCCATCCACATCCTCCTGC	150 bp
		R: GCTGCTGTAGAAGCCGAAAC	
*GPR84*	NM_020370.2	F: ACCGCCAGGTCAAACGAG	157 bp
		R: ATCCCCTCACTGGGTCCTC	
*GPR119*	NM_178471.2	F: GCCGTGTTTCACCCTCG	207 bp
		R: CACAGTTCGGACAGCCTTG	
*GPR120*	NM_001204766.1	F: CGTTTCCCGTTCTTCTCCG	100bp
		R: CCAGCAGCGACACCACAAA	
*ACC*	AF175308	F: CTCCAGGACAGCACAGATCA R: GCCGAAACATCTCTGGGATA	170 bp
*FAS*	EF589048	F: GGACCTGGTGATGAACGTCT R:CGGAAGTTGAGGGAGGTGTA	225 bp
*ACO*	AF185048	F: CTCGCAGACCCAGATGAAAT R: TCCAAGCCTCGAAGATGAGT	218 bp
*FABP*	DQ182323	F: TTCGGTGCATGTCTAAGCTG R: TGAGAGGGAGAGGATGAGGA	200 bp
*SREBP-1*	NM_214157	F: CCTCTGTCTCTCCTGCACC R: ACAAAGAGAAGCGCCAAGAA	213 bp
*SREBP-1c*	AY307771	F: CCTCTGTCTCTCCTGCAACC R: GACCGGCTCTCCATAGACAA	229 bp
*TGH*	NM_214246.1	F: TACATCGTGGGAATCAACAAG R: GCTTGGGCGATACTGAAAC	325 bp
*ATGL*	NM_001098605.1	F: ATGGTGCCCTACACGCTG R: GCCTGTCTGCTCCTTTATCC	111 bp

### Statistical analyses

The data of gene expression were showed as means ± SEM, and FFA concentrations were shown as means ± standard deviation (SD). Statistical analysis was performed using a 2 × 2 between-subjects factorial design analysis of variance (ANOVA), and chi-square tests for each relevant variable. Statistical analyses were performed using SAS 9.2 (SAS Institute Inc., Cary, NC). The differences were considered as statistically significant for *P* < 0.05.

## Results

### MSG and/or fat did not obviously influence the growth performance and carcass composition of growing pigs

Based on our published research (Table 3) (47, 48), no obvious effects were found on the growth performance and carcass from dietary supplementation with MSG and/or fat.

**Table 3:** Effect of dietary MSG and/or fat on the growth performance of growing pigs (*n* = 8) (47, 48)

Item	Measurements (Mean ± SE)				Analysis of variance(*P*)		
	BD	HF	BDM	HFM	Fat effect	MSG effect	Interaction
Feed intake (kg/d)	2.07 ± 0.09	1.78 ± 0.09	1.98 ± 0.11	1.92 ± 0.08	0.77	0.08	0.23
Daily gain (kg)	0.80 ± 0.05	0.77 ± 0.06	0.80 ± 0.05	0.76 ± 0.07	0.89	0.52	0.88
Feed conversion ratio	2.67 ± 0.08	2.40 ± 0.13	2.55 ± 0.03	2.66 ± 0.14	0.51	0.16	0.10
Total skeletal muscle (%)	61.02 ± 2.70	61.63 ± 1.71	59.08 ± 0.68	61.66 ± 0.96	0.37	0.59	0.57
Total fat (%)	13.34 ± 2.94	15.49 ± 0.48	16.29 ± 0.35	14.87 ± 1.24	0.82	0.49	0.29
Total bone (%)	17.04 ± 0.31	14.04 ± 1.36	15.84 ± 1.07	14.22 ± 0.68	0.03	0.59	0.48
Total skin (%)	8.6 ± 0.38	8.84 ± 0.14	8.79 ± 0.23	9.25 ± 0.50	0.33	0.39	0.75

### MSG and/or fat promoted the fat deposition of growing pigs

**Fig. 1:**
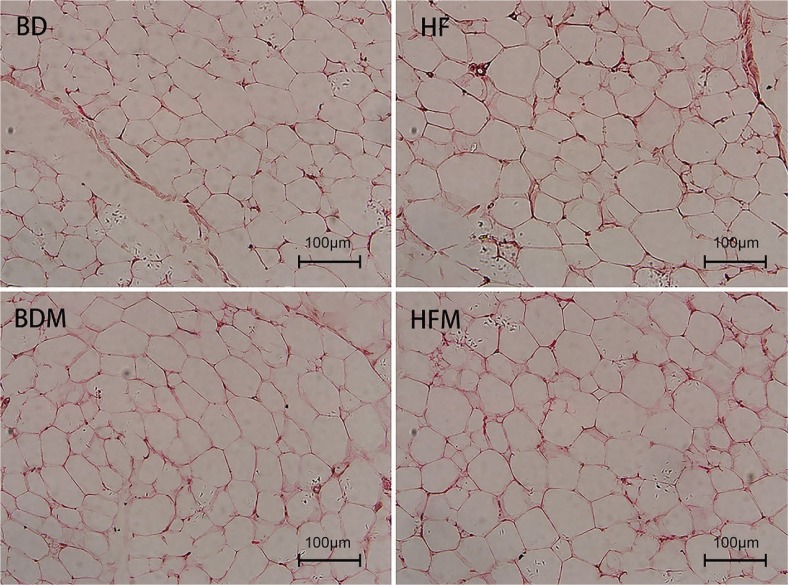
Effect of dietary MSG and/or fat on the morphological of back adipose tissue.

In the present study, MSG or fat could obviously promote adipocytes volume in back adipose tissue, while the addition of fat and MSG together had an antagonistic effect on the size of fat cells (Fig. 1). Adipocytes size comparison showed that dietary addition of fat and MSG can promote fat deposition.

### MSG and fat exhibited little effect on total FFAs in plasma and hypothalamic homogenate of growing pigs

**Fig. 2:**
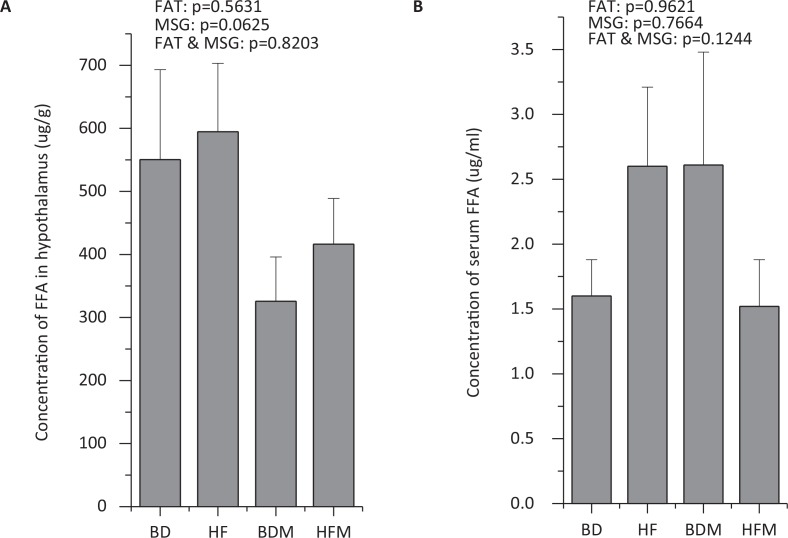
Effect of dietary MSG and/or fat on total FFA concentration in plasma and hypothalamus (*n ≥ 4*).

In the present study, the concentrations of FFA in blood and hypothalamic homeostasis were determined. However, no obvious effects were found from MSG and/or fat on total FFAs in plasma and hypothalamus (Fig. 2).

### MSG and/or fat affected SCFA concentrations in intestinal luminal contents

In the present study, SCFA levels in cecum and colonic contents were determined to test the effect of MSG and/or fat. The results showed that fat significantly increased (*P* < 0.05) the concentration of isobutyrate and isovalerate in cecum contents (Table 4), and simultaneously remarkably decreased (*P* < 0.05) valerate level in colonic contents (Table 5). MSG significantly elevated (*P* < 0.05) the butyrate levels in colonic contents (Table 5). MSG and fat alone or in combination showed no significant effect on the ratio of propionate/acetate in cecal and colonic contents (Tables 4 and 5). No obvious interactive effects were found on intestinal luminal SCFA concentrations when MSG and fat were supplemented together.

**Table 4:** Effect of dietary MSG and/or fat on SCFAs concentration in cecum contents (*n ≥ 4*)

SCFAs	Measurements (Mean ± SD) mg/g				Analysis of variance *(P*)		
	BD	HF	BDM	HFM	Fat Effect	GSM Effect	Interaction
Acetate	9.5037 ± 1.0647	15.1083 ± 1.7796	14.3999 ± 4.1425	9.3923 ± 3.4759	0.9196	0.8898	0.0918
Propionate	6.7412 ± 1.1300	8.9725 ± 0.5778	8.2588 ± 2.1020	6.5713 ± 1.9885	0.8662	0.7845	0.2386
Isobutyrate	0.3795 ± 0.0227	0.4946 ± 0.0421	0.3855 ± 0.0342	0.5009 ± 0.0880	***0.0427***	0.9035	0.998
Butyrate	4.0305 ± 0.7341	6.5575 ± 1.2708	6.6041 ± 1.6583	4.2746 ± 1.3383	0.9404	0.9124	0.085
Isovalerate	0.3715 ± 0.0348	0.5358 ± 0.0601	0.4363 ± 0.0452	0.6032 ± 0.1253	***0.0321***	0.337	0.984
Valerate	0.7272 ± 0.0810	1.0798 ± 0.1347	1.0372 ± 0.0998	1.0696 ± 0.2450	0.2348	0.3493	0.3186
Total SCFAs	21.6587 ± 2.3088	32.7484 ± 3.4437	31.1218 ± 7.9523	22.1359 ± 7.1056	0.8572	0.9216	0.1048
Propionate/acetate	0.7335 ± 0.1628	0.6106 ± 0.0576	0.6000 ± 0.0500	0.7417 ± 0.1430	0.936	0.9918	0.2719

Bold values indicate statistically significant (P < 0.05).

**Table 5:** Effect of dietary MSG and/or fat on SCFAs concentration in colon contents (*n ≥ 4*)

SCFAs	Measurements (Mean ± SD) mg/g				Analysis of variance (*P*)		
	BD	HF	BDM	HFM	Fat effect	GSM effect	Interaction
Acetate	10.7245 ± 1.3460	7.4059 ± 1.4107	8.0427 ± 0.2981	9.0766 ± 0.6201	0.2909	0.6337	0.057
Propionate	6.7936 ± 0.9031	4.8653 ± 0.7113	6.6996 ± 0.7114	6.1926 ± 0.2344	0.1012	0.3864	0.3207
Isobutyrate	0.6534 ± 0.0816	0.5498 ± 0.0374	0.6186 ± 0.0453	07563 ± 0.0576	0.7734	0.1638	0.0592
Butyrate	5.0858 ± 0.6725	4.4380 ± 0.5777	7.4972 ± 0.4863	6.7379 ± 0.4971	0.2356	***0.0013***	0.9228
Isovalerate	0.7860 ± 0.1188	0.5616 ± 0.0577	0.7004 ± 0.0750	0.8497 ± 0.0988	0.686	0.286	0.0615
Valerate	1.1964 ± 0.1827	0.9732 ± 0.0647	1.5025 ± 0.0816	1.1875 ± 0.1280	***0.0493***	0.0561	0.7157
Total SCFAs	25.2397 ± 3.0661	18.7939 ± 2.6156	25.0610 ± 1.3007	24.8006 ± 0.9617	0.1485	0.2044	0.1798
Propionate/acetate	0.6321 ± 0.0104	0.6737 ± 0.0534	0.8280 ± 0.0632	0.6939 ± 0.0607	0.3872	0.0581	0.1144

Bold values indicate statistically significant (P < 0.05).

### MSG and/or fat affected LCFA compositions in intestinal luminal contents

In the present study, dietary fat significantly increased (*P* < 0.05) the percentages of myristic acid, linoleic acid, linolenic acid, behenic acid and eicosatrienoic acid in ileac contents, whereas it remarkably decreased (*P* < 0.05) the percentages of palmitoleic acid, stearic acid and eicosapentaenoic acid. Conversely, MSG significantly increased (*P* < 0.05) the percentages of myristic acid, stearic acid and elaidic acid and decreased (*P* < 0.05) the percentages of palmitoleic acid, linoleic acid, linolenic acid, arachidic acid and tetracosanoic acid in ileac contents. Apart from synergistically increased (*P* < 0.05) the percentage of myristic acid, MSG and fat exhibited antagonistic effect (*P* < 0.05) on palmitoleic acid, heptadecanoic acid, stearic acid, linoleic acid and arachidonic acid in ileum contents (Table 6).

**Table 6:** Effect of dietary MSG and fat and its interaction on the concentrations of LCFAs in ileum contents (*n ≥ 4*)

LCFAs	Measurements (Mean ± SD) %				Analysis of variance (*P*)		
	BD	HF	BDM	HFM	Fat effect	GSM effect	Interaction
Myristic acid	0.1765 ± 0.0150	0.2000 ± 0.0217	0.2260 ± 0.0450	0.3840 ± 0.0723	***0.0014***	***0.0002***	***0.0088***
Palmitic acid	17.3697 ± 2.2662	16.4697 ± 1.1418	18.5180 ± 1.1864	21.2463 ± 8.9575	0.7712	0.1152	0.2854
Palmitoleic acid	0.3792 ± 0.0378	0.2775 ± 0.0146	0.2855 ± 0.0559	0.2773 ± 0.0280	***0.0055***	***0.0174***	***0.0232***
Heptadecanoic acid	0.2565 ± 0.0058	0.2528 ± 0.0356	0.2440 ± 0.0404	0.3125 ± 0.0364	0.0705	0.1732	***0.0469***
Stearic acid	6.5517 ± 0.9030	6.3143 ± 1.3101	6.7323 ± 1.0911	11.2123 ± 1.8896	***0.0060***	***0.0006***	***0.0003***
Oleic acid	23.3741 ± 1.2695	23.1510 ± 0.7655	21.9117 ± 0.7488	21.0088 ± 2.1190	0.4443	***0.0032***	0.5253
Elaidic acid	1.2716 ± 0.2646	1.1422 ± 0.1487	1.5325 ± 0.2635	1.8363 ± 0.9158	0.8774	***0.0217***	0.2439
Linoleic acid	46.6323 ± 3.9153	46.9215 ± 3.6134	46.1833 ± 2.6880	36.3940 ± 5.7206	***0.0362***	***0.0097***	***0.0071***
Arachidic acid	0.5041 ± 0.1366	0.5105 ± 0.0920	0.3962 ± 0.0351	0.4200 ± 0.0752	0.6285	***0.0232***	0.8342
Linolenic acid	1.2036 ± 0.1550	1.4747 ± 0.1585	1.0932 ± 0.2112	1.4645 ± 0.2402	***0.0007***	0.4016	0.5373
Behenic acid	0.3912 ± 0.1178	0.5340 ± 0.1770	0.3028 ± 0.0452	0.6000 ± 0.1740	***0.0065***	0.9032	0.2659
Eicosatrienoic acid	0.7158 ± 0.2126	0.8495 ± 0.1551	0.6925 ± 0.1795	1.0783 ± 0.1645	***0.0075***	0.3345	0.1569
Arachidonic acid	0.8100 ± 0.2256	0.5245 ± 0.1172	0.5445 ± 0.0186	0.6248 ± 0.0641	0.1444	0.2326	***0.0166***
Eicosapentaenoic acid	0.9443 ± 0.2214	0.6625 ± 0.0816	0.8262 ± 0.1194	0.6998 ± 0.2248	***0.0326***	0.5998	0.3657
Tetracosanoic acid	0.3625 ± 0.0533	0.4625 ± 0.0788	0.2492 ± 0.0637	0.3005 ± 0.1249	0.0979	***0.0087***	0.8742

Bold values indicate statistically significant (P < 0.05).

Similar to ileum, dietary fat and MSG affected cecal contents LCFAs composition and relative proportion. As shown in Table 7, dietary fat significantly increased (*P* < 0.05) the percentages of pentadecanoic acid, linoleic acid, linolenic acid, eicosadienoic acid and eicosatrienoic acid and remarkably decreased (*P* < 0.05) oleic acid and arachidonic acid percentages in cecal contents. However, MSG remarkably elevated (*P* < 0.05) the percentages of palmitoleic acid, linoleic acid, eicosadienoic acid and eicosapentaenoic acid, whereas it decreased (*P* < 0.05) palmitic acid, stearic acid, oleic acid and behenic acid percentages in cecal contents. Simultaneously, MSG and fat exhibited antagonistic effect (*P* < 0.05) on the percentages of palmitic acid, palmitoleic acid, heptadecanoic acid, stearic acid, elaidic acid, linoleic acid, arachidic acid and arachidonic acid in cecal contents.

**Table 7:** Effect of dietary MSG and fat and its interaction on the concentrations of LCFAs in cecum contents (*n ≥ 4*)

LCFAs	Measurements (Mean ± SD) %				Analysis of variance (*P*)		
	BD	HF	BDM	HFM	Fat effect	GSM effect	Interaction
Myristic acid	1.3705 ± 0.3581	1.2900 ± 0.3910	1.5097 ± 0.3413	1.6703 ± 0.2750	0.7411	0.0765	0.3768
Pentadecanoic acid	2.1771 ± 0.4389	3.9003 ± 0.9347	2.8023 ± 0.5959	3.3683 ± 1.3523	***0.0019***	0.7437	0.0959
Palmitic acid	21.7126 ± 2.6667	18.3292 ± 1.7148	16.1028 ± 1.8735	20.2170 ± 1.9049	0.6548	***0.0062***	***0.0001***
Palmitoleic acid	0.4726 ± 0.1511	0.6408 ± 0.1059	1.0776 ± 0.2352	0.6672 ± 0.1040	0.1133	***0.0004***	***0.0009***
Heptadecanoic acid	0.6126 ± 0.1268	0.5238 ± 0.0522	0.4872 ± 0.2347	0.7162 ± 0.1439	0.2048	0.9272	***0.0349***
Stearic acid	8.5571 ± 1.3408	4.9655 ± 0.9249	4.0063 ± 0.7597	6.4392 ± 1.6543	0.2184	***0.0002***	***<0.0001***
Oleic acid	24.6335 ± 1.8776	21.6382 ± 1.5668	22.5045 ± 0.6665	21.4110 ± 1.7343	***0.0017***	***0.0308***	0.1133
Elaidic acid	2.0709 ± 0.4414	1.7968 ± 0.1481	1.5410 ± 0.2839	2.2403 ± 0.5225	0.1171	0.2975	***0.0059***
Linoleic acid	30.3954 ± 4.1876	32.7188 ± 4.8027	40.7716 ± 3.8014	29.8168 ± 3.9442	***0.0126***	***0.0064***	***0.0004***
Arachidic acid	0.4259 ± 0.0770	0.4190 ± 0.0338	0.3825 ± 0.0489	0.5128 ± 0.1019	0.0501	0.6877	***0.0415***
Linolenic acid	1.4661 ± 0.3251	1.7348 ± 0.4119	1.1206 ± 0.3524	1.8140 ± 0.1562	***0.0013***	0.1905	0.1183
Eicosadienoic acid	0.4163 ± 0.1087	1.2788 ± 0.3057	1.1310 ± 0.0765	1.7805 ± 0.4539	***0.0002***	***0.0010***	0.4639
Behenic acid	0.6323 ± 0.2062	0.8163 ± 0.2846	0.4556 ± 0.2344	0.4033 ± 0.0522	0.2418	***0.0085***	0.2312
Eicosatrienoic acid	1.7930 ± 0.4593	2.6188 ± 0.6647	1.4855 ± 0.5746	2.3925 ± 0.7518	***0.0036***	0.3339	0.8829
Arachidonic acid	1.7308 ± 0.1543	0.5955 ± 0.0722	1.0813 ± 0.1028	1.4633 ± 0.1612	***<0.0001***	0.1140	***<0.0001***
Eicosapentaenoic acid	0.8960 ± 0.2928	1.2755 ± 0.1677	1.5300 ± 0.6615	1.6203 ± 0.3411	0.1630	***0.0043***	0.3846
Tetracosanoic acid	0.5080 ± 0.2156	0.5223 ± 0.0796	0.5505 ± 0.1594	0.4690 ± 0.0754	0.5962	0.9795	0.4908

Bold values indicate statistically significant (P < 0.05).

As shown in Table 8, high dietary fat significantly elevated (*P* < 0.05) the percentages of myristic acid, pentadecanoic acid, stearic acid, eicosadienoic acid, eicosatrienoic acid and eicosapentaenoic acid, but decreased (*P* < 0.05) the percentages of palmitic acid, elaidic acid, linoleic acid and arachidic acid in colonic contents. MSG remarkably increased (*P* < 0.05) the percentages of myristic acid, linoleic acid, linolenic acid, eicosadienoic acid, eicosatrienoic acid and tetracosanoic acid, while it decreased (*P* < 0.05) the percentages of pentadecanoic acid, palmitic acid and oleic acid in colonic contents. Besides synergistic elevation (*P* < 0.05) of myristic acid, MSG and fat exhibited antagonistic effect (*P* < 0.05) on the percentages of palmitic acid, palmitoleic acid, stearic acid, elaidic acid, linoleic acid, behenic acid and eicosatrienoic acid in colonic contents.

**Table 8:** Effect of dietary MSG and fat and its interaction on the concentrations of LCFAs in colon contents (*n ≥ 4*)

LCFAs	Measurements (Mean ± SD) %				Analysis of Variance (*P*)		
	BD	HF	BDM	HFM	Fat effect	GSM effect	Interaction
Myristic acid	1.5233 ± 0.1327	2.4970 ± 0.1750	2.6813 ± 0.3893	7.1018 ± 1.2835	***<0.0001***	***<0.0001***	***0.0003***
Pentadecanoic acid	3.3465 ± 0.2815	6.3575 ± 0.6124	1.9395 ± 0.2406	4.9428 ± 0.5372	***<0.0001***	***<0.0001***	0.9865
Palmitic acid	23.4653 ± 0.8167	23.3563 ± 0.8243	18.2265 ± 2.2905	21.7278 ± 1.3296	***0.0370***	***0.0005***	***0.0281***
Palmitoleic acid	0.6960 ± 0.1619	0.6293 ± 0.0224	0.4373 ± 0.0784	0.6840 ± 0.1170	0.1211	0.0830	***0.0132***
Heptadecanoic acid	0.7070 ± 0.2542	0.7670 ± 0.1927	0.6238 ± 0.1815	0.9195 ± 0.0507	0.0789	0.7150	0.2272
Stearic acid	4.2995 ± 0.8443	5.5690 ± 0.9506	3.6268 ± 0.7152	7.0330 ± 0.3715	***<0.0001***	0.3138	***0.0149***
Oleic acid	26.1005 ± 1.2541	24.3600 ± 2.9086	21.9140 ± 0.7122	21.9245 ± 1.7259	0.3652	***0.0036***	0.3596
Elaidic acid	1.7838 ± 0.3318	1.1838 ± 0.1551	1.4098 ± 0.1426	1.2995 ± 0.1573	***0.0057***	0.2460	***0.0392***
Linoleic acid	24.4550 ± 1.1431	22.4450 ± 2.9937	36.3580 ± 4.0934	18.1935 ± 1.7802	***<0.0001***	***0.0165***	***<0.0001***
Arachidic acid	0.5355 ± 0.0639	0.3870 ± 0.0499	0.6420 ± 0.0448	0.4270 ± 0.1311	***0.0007***	0.0929	0.4234
Linolenic acid	1.4420 ± 0.0641	1.4008 ± 0.1018	1.8380 ± 0.1692	1.6273 ± 0.2424	0.1402	***0.0021***	0.3090
Eicosadienoic acid	0.1860 ± 0.0046	0.5030 ± 0.0173	0.4685 ± 0.0623	0.6238 ± 0.2283	***0.0018***	***0.0053***	0.1980
Behenic acid	0.8848 ± 0.2711	0.7268 ± 0.0565	0.6373 ± 0.0229	0.9203 ± 0.1773	0.3480	0.9148	***0.0302***
Eicosatrienoic acid	0.8567 ± 0.1104	3.2460 ± 0.7170	2.1943 ± 0.3946	3.1878 ± 0.1296	***<0.0001***	***0.0099***	***0.0059***
Eicosapentaenoic acid	0.8660 ± 0.0796	1.5728 ± 0.3063	0.7960 ± 0.1399	1.7785 ± 0.4445	***<0.0001***	0.6385	0.3469
Tetracosanoic acid	0.4505 ± 0.0605	0.4193 ± 0.0560	0.5018 ± 0.1003	0.6513 ± 0.1515	0.2588	***0.0149***	0.0951

Bold values indicate statistically significant (P < 0.05).

### MSG and/or fat downregulated FFA receptors gene expression in gastrointestinal tract

**Fig. 3:**
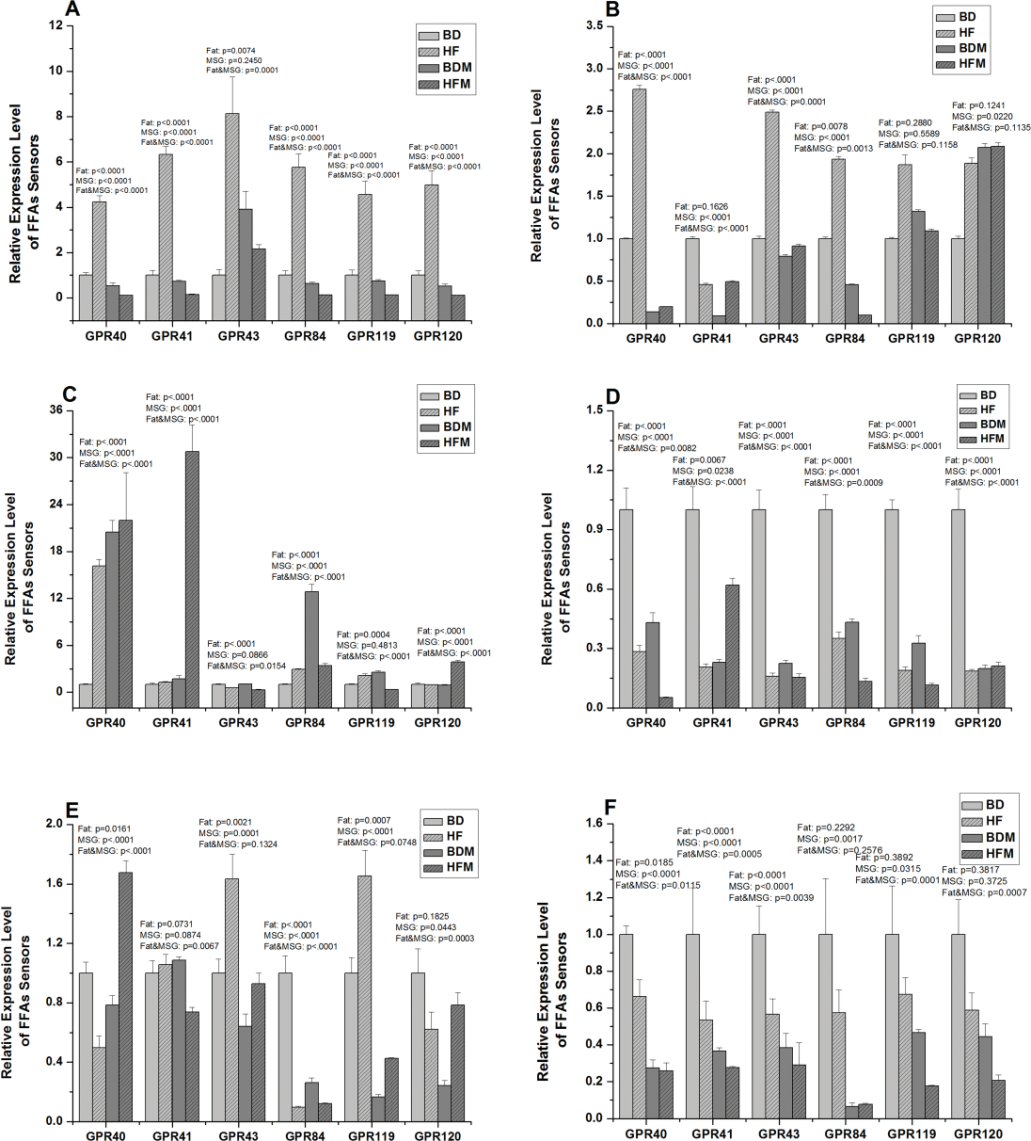
Effect of dietary MSG and/or fat on FFA sensors expression in hypothalamus and gastrointestinal tract (*n ≥ 4*). A, stomach; B, duodenum; C, jejunum; D, ileum; E, colon; F, hypothalamus. Data are presented as mean ± standard error.

In the present study, MSG and/or fat were also found to change the concentrations and relative proportion of FFAs in intestinal luminal contents; thus, we investigated whether MSG and/or fat may change the expression profiles of corresponding FFA receptors (GPR40, GPR41, GPR43, GPR84, GPR119 and GPR120) in gastrointestinal tract. As shown in Fig. 3, high dietary fat significantly increased (*P* < 0.05) all tested FFA receptors expression. However, besides elevation of GPR43 expression, MSG remarkably decreased (*P* < 0.05) other FFA receptors expression in stomach. Simultaneously, MSG and fat combination significantly decreased (*P* < 0.05) FFA receptors expression except for GPR43 and exhibited antagonistic effect in stomach.

Apart from significant elevation (*P* < 0.05) of GPR40, GPR43 and GPR84 expression in fat treatment group, both MSG and fat significantly decreased (*P* < 0.05) GPR40, GPR41, GPR43 and GPR84 expression. At the same time, MSG and fat combination exhibited antagonistic effect on FFA receptors expression in duodenum. However, all treatment groups exhibited no effect on GPR119 and GPR120 expression (Fig. 3B) in duodenum.

As shown in Fig. 3C, except for significantly decreased (*P* < 0.05) expression of GPR43, dietary fat remarkably increased (*P* < 0.05) other FFA receptors gene expression in jejunum. MSG significantly elevated (*P* < 0.05) the expression of GPR40, GPR41, GPR84 and GPR120 in jejunum. At the same time, MSG and fat combination exhibited synergistic effect on gene expression of GPR40, GPR41 and GPR120 and antagonistic effect on GPR43, GPR84 and GPR119 expression in jejunum.

Interestingly, both dietary fat and MSG significantly decreased (*P* < 0.05) all the FFA receptors expression in ileum. Simultaneously, MSG and fat combination exhibited synergistic effect on the gene expression of GPR40, GPR84 and GPR119, but antagonistic effect on GPR41, GPR43 and GPR120 expression in ileum (Fig. 3D).

As shown in Fig. 3E, high dietary fat significantly decreased (*P* < 0.05) the gene expression of GPR40 and GPR84, while it increased (*P* < 0.05) GPR43 expression in colon. Besides GPR41, MSG significantly decreased (*P* < 0.05) FFA receptors expression in colon. At the same time, dietary fat and MSG combination exhibited antagonistic effect on GPR40, GPR41, GPR84 and GPR120 expression in colon.

Collectively, MSG and/or fat exhibit different effects on FFA receptors and vary with its segments and compartments. Simultaneously, MSG and fat exhibit antagonistic effect on most of FFA receptors.

### MSG and/or fat downregulated FFA receptors expression in hypothalamus

As shown in Fig. 3F, high dietary fat remarkably decreased (*P* < 0.05) the expression of GPR40, GPR41 and GPR43. Similarly, MSG significantly decreased (*P* < 0.05) FFA receptors gene expression except GPR120. Interestingly, besides GPR84, MSG and fat combination synergistically decreased other FFA receptors gene expression in hypothalamus. Collectively, MSG and/or fat blunted FFAs sensing ability through downregulation of FFA receptors in hypothalamus.

### MSG and/or fat affect fatty acid metabolism in back fat

**Fig. 4:**
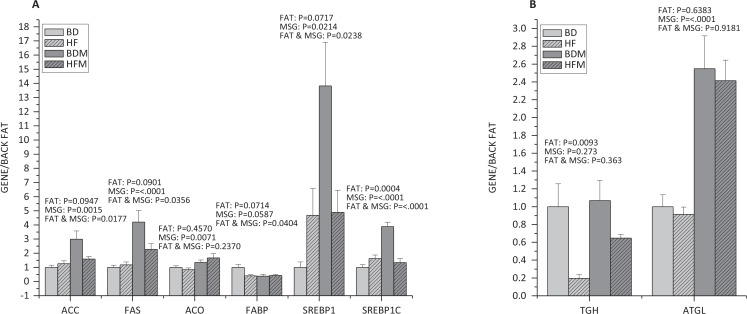
Effect of dietary MSG and/or fat on the expression of genes related to fatty acid metabolism in back fat (*n ≥ 4*). A, the expression of genes related to fat synthesis; B, the expression of genes related to adipose decompose. Data are presented as mean ± standard error.

As shown in Fig. 4A, high dietary fat remarkably increased (*P* < 0.05) the expression of SREBP-1c, while MSG significantly increased (*P* < 0.05) the expression of acetyl-CoA carboxylase (ACC) (*P* < 0.05), FAS (*P* < 0.0001), acyl-CoA oxidase (ACO) (*P* < 0.05), SREBP-1 (*P* < 0.05) and SREBP-1c (*P* < 0.0001). Fat and MSG together had antagonistic effect on the expression of ACC (*P* < 0.05), FAS (*P* < 0.05), FABP (*P* < 0.05), SREBP-1 (*P* < 0.05) and SREBP-1c (*P* < 0.0001).

Compared to the mRNA levels of genes related to fat synthesis, dietary fat remarkably decreased the expression of triacylglycerol hydrolase (TGH) (*P* < 0.05) related to adipose decompose, while MSG significantly increased the expression of adipose triglyceride lipase (ATGL) (*P* < 0.0001) (Fig. 4B).

## Discussion

Intestinal luminal FFAs, especially in colon, are mainly derived from anaerobic bacterial fermentation of dietary carbohydrates. Except for producing ATP for enterocytes, FFAs modulate physiological and immune functions. However, over-intake of FFAs or high concentration of circulating FFAs is strongly associated with metabolic syndromes, such as obesity (9). To prevent this negative effect, FFA receptors have been evolved to regulate the absorption and biological role of FFAs. Many factors, including dietary composition, affect the FFA levels in intestinal luminal contents and FFA receptors expression profiles in gastrointestinal tract. Previous studies had shown that MSG and/or fat affected circulating amino acid pool (46), intestinal barrier function, immunity (49) and intestinal microbiota (44) in growing pigs; however, less study focus was given on the effect of MSG and/or fat alone, or in combination, on FFA levels in intestinal luminal contents and the expression of FFA receptors in gastrointestinal tract. In the present study, we reported, for the first time, that MSG and/or fat affect the FFA levels in intestinal luminal contents and expression profiles of FFA receptors in hypothalamus and gastrointestinal tract of growing pigs.

Collectively, our data support that MSG and fat (alone and in combination) significantly affect the composition and relative proportion of LCFAs in intestinal luminal contents and simultaneously exhibit antagonistic effect.

The SCFAs from microbial fermentation in colon provide approximately 10% extra energy of diet, and this energy can be used for *de novo* synthesis of triglycerides and gluconeogenesis (50, 51). Although FFAs could be beneficial to some extent, excessive FFAs uptake or higher circulating FFAs levels increase the risk to induce insulin resistance and obesity and other metabolic syndromes (52, 53). SCFAs mainly include acetate, propionate, butyrate and valerate. Acetate is a necessary energy resource for muscle cell, cardiomyocytes and brain cells (54); propionate can be used for gluconeogenesis (55) and butyrate is an important energy supplier for intestinal epithelial cells (56). The ratio of acetate to propionate is usually regarded as a marker to reflect the energy status in the body (57). In the present study, MSG and fat alone or in combination exhibited no significant effect on the ratio of acetate to propionate and most of SCFA concentrations in the cecum and colonic contents. Although fat could increase the isobutyric acid and isovaleric acid concentration in cecum contents, they showed less effect on the body than other SCFAs. Interestingly, MSG elevated butyrate concentration in cecum and colonic contents. MSG remarkably enhanced colonisation of butyrate-producing bacteria in colon (44), which ferment fibre to produce butyrate (58). This may explain why MSG increases butyrate level in the colonic contents. Notably, SCFAs, especially butyrate regulate proliferation and differentiation of intestinal epithelial cells, enhance intestinal barrier function, and additionally modulate inflammatory response (59, 60). At the same time, dietary supplementation with SCFAs improves energy metabolic homeostasis (61). However, based on previous studies, high butyrate and acetate level in colon is strongly associated with pathogenesis of obesity (62, 63); therefore, it is difficult to evaluate the effect of MSG and/or fat on obesity development only according to intestinal luminal SCFAs concentration. Thus, more works should be carried out to elucidate it clearly.

Unlike SCFAs, LCFAs are absorbed through lymphatic vessels in a chyle manner. Emerging evidences have shown that the length of carbon chain of FFAs affects the absorption and metabolism of fatty acid and the gene expression profiles (64). Actually, over-uptake of LCFAs is a major contributor to obesity, specifically triglycerides accumulation in adipose and other tissues because of uptake of LCFAs in adipocytes (65). Hence the advances in revealing the association of MSG and/or fat with LCFAs composition and percentages in intestinal luminal contents can deepen the understanding of the relationship of MSG and fat with obesity. In the present study, MSG and fat alone or in combination significantly affected intestinal luminal LCFAs composition. The main fat source in this study was soybean oil which contains large amounts of unsaturated fatty acids (Table 1); therefore, the dietary fat addition could increase polyunsaturated fatty acids and decrease saturated fatty acids in intestinal luminal. Among these fatty acids, dietary fat mainly increased linoleic acid and linolenic acid (polyunsaturated fatty acids) percentages and decreased stearic acid, myristic acid and palmitic acid (saturated fatty acids) percentages in ileum and cecum. On the contrary, MSG increased some of saturated fatty acids percentage and decreased some of polyunsaturated fatty acids percentage in ileum and cecum. Simultaneously, MSG and fat exhibit antagonistic effect. However, the effects of MSG and fat on LCFAs in colon are complicated. Linoleic acid and oleic acid are two main unsaturated fatty acids in the diet, which were reported to be beneficial to health (66). Linolenic acid is also beneficial to the body although it is relatively rare in the diet. α-Linolenic acid can effectively alleviate coronary artery disease and reduce mortality (67, 68). γ-Linolenic acid can significantly reduce weight, which can be used in the treatment of obesity (69−70). Palmitic acid and stearic acid are the two most saturated types of dietary LCFA (71). Palmitic acid, instead of unsaturated oleic acid and linoleic acid, can lead to the secretion of inflammatory cytokines and induce neurotoxicity through the activation of signalling pathways including JNK (72). Other saturated fatty acids such as lauric acid, cardamic acid and palmitic acid promote the accumulation of triacylglycerol in cells and produce ROS (73). Dietary fat promoted the absorption of these beneficial unsaturated fatty acids for body, while MSG had a relatively weak effect. Both fat and MSG in dietary supplement could produce beneficial effects on the body by effectively reducing the amount of palmitate in the intestinal tract, while dietary supplementation with MSG showed an opposite effect to fat on the main saturated acids (palmitic acid) as it does on the main unsaturated fatty acids (linoleic acid and oleic acid). This result indicated that the dietary addition of MSG has more harmful effects on the body than fat. Thus, MSG and fat alone or in combination affected intestinal luminal lipid metabolism and intestinal health by changing intestinal luminal LCFAs composition and concentration, finally exerting different effect on development of obesity.

Previous studies have demonstrated that the role of FFAs in human health is sensed and regulated by FFA receptors (74), and these FFA receptors play an essential role in lipid and energy metabolism, insulin resistance, fat accumulation, and even pathogenesis of obesity and diabetes (14, 28, 29). FFA receptors are divided into SCFA receptors (GPR41 and GPR43) (23) and LCFA receptors (GPR40, GPR84 and GPR120) (22, 27) and then regulate the corresponding FFAs biological function, respectively. Additionally, SCFAs activate GPR41 and GRP43 cascade signal pathways, which lead the release of gastrointestinal hormone peptide YY, and further regulate energy metabolism (75). According to previous study of diet-induced obese rat, blunt of nutrients-sensing is an important factor for obesity development (76). Consistent with previous investigation, MSG and/or fat significantly downregulate FFA receptors expression in ileum and hypothalamus. At the same time, MSG and fat in combination exhibit synergistic effect. Ileum is a key site for FFAs absorption, and hypothalamus is a control centre of energy metabolism (77). Thus, the suppression of FFA receptors in ileum and hypothalamus may cause dysfunction of sensing and surveillance of lipid, and effect the development of obesity (28, 74). However, dietary fat significantly increased gene expression of FFA receptors, including SCFAs and LCFAs receptors in stomach, duodenum and jejunum. As enzymatic hydrolysis of fat in diet, most of FFAs are released from dietary fat in the proximal and middle intestine. Therefore, increased expression of FFA sensors expression may facilitate luminal FFAs flux absorption and metabolism (14). Intriguingly, dietary fat increases gene expression of SCFA receptors and conversely decreases gene expression of LCFA receptors in colon. The majority of SCFAs are produced in colon because colon is the major site for microbial fermentation of carbohydrates including dietary fibre (11). Simultaneously, SCFAs prefer to be oxidised to produce ATP for colonocyte (56). Thus, upregulation of SCFA receptors expression facilitates SCFAs absorption and metabolism. However, as the LCFAs concentration in colon factually increases, reduced expression of LCFA receptors in colon may lead to dysfunctional regulation of LCFAs metabolism. Of note is that LCFAs are the critical contributor for fat accumulation and obesity (65). According to previous studies, we know that FFA receptors have come to be regarded as new drug targets for metabolic disorder, such as obesity and type 2 diabetes (78). Such dysfunction of GPR120 could be a potential mechanism for HFD-induced obesity and obesity-associated metabolic syndrome in mice (28), and the expression of GPR120 in X/A-like cells causes a reduced level of ghrelin (79−81), suggesting that the activation of GPR120 has an anti-obesity effect. Consequently, the suppression of LCFA receptors induced by fat may be a candidate mechanism for obesity development. Dietary MSG decreased most FFA sensors expression, and this may explain why MSG induces obesity. However, further detailed studies such as molecular interactions and signalling pathways are needed to elucidate the relationship between FFA receptors and obesity in condition of MSG and/or fat diet. Collectively, MSG and/or fat blunted FFA sensors in hypothalamus and gastrointestinal tract, possibly leading to dysfunctional regulation of FFAs and energy metabolism, which may link with the development of obesity.

Although MSG and/or fat had no significant effects on the growth performance of growing pigs, which may be due to the short feeding period, however, they significantly increased intramuscular lipid content by promoting fat synthesis (47). Similarly, the present study showed increased lipid content in adipose tissue (Fig. 1). The most direct symptom of obesity is the increase of body fat rate. When the balance of fat metabolism was disturbed, it resulted in imbalance of fat synthesis and decomposition, and the increase of net fat deposition will eventually lead to obesity (82). In the present study, MSG increased the fat synthesis and decomposition, while the synthesis rate is higher than the decomposition rate, leading to the deposition of fat. Fat increases the fat synthesis and reduces fat decomposition, resulting in the deposition of fat. The effects of MSG and fat in combination on fat metabolism of growing pigs were antagonistic.

## Conclusion

MSG and/or fat significantly affect intestinal luminal FFAs concentration, especially LCFAs. The effect of MSG and fat on LCFAs composition and concentration in intestinal luminal contents is complicated and different, and simultaneously exhibits antagonistic effect, which may represent the different mechanisms of MSG and fat on the effect of obesity development. Importantly, MSG and/or fat significantly blunted FFA receptors expression in hypothalamus and gastrointestinal tract, and suppression of FFAs sensors may cause dysfunctional metabolism of lipid and energy. Thus, downregulation of FFA receptors expression induced by MSG and fat may be one of the mechanisms for obesity development. Furthermore, MSG and/or fat promoted fat deposition in adipose tissue through different ways.
